# Calpain-Catalyzed Proteolysis of Human dUTPase Specifically Removes the Nuclear Localization Signal Peptide

**DOI:** 10.1371/journal.pone.0019546

**Published:** 2011-05-19

**Authors:** Zoltán Bozóky, Gergely Róna, Éva Klement, Katalin F. Medzihradszky, Gábor Merényi, Beáta G. Vértessy, Peter Friedrich

**Affiliations:** 1 Institute of Enzymology, Hungarian Academy of Sciences, Budapest, Hungary; 2 Proteomics Research Group, Biological Research Centre (BRC), Hungarian Academy of Sciences, Szeged, Hungary; 3 Department of Pharmaceutical Chemistry, University of California San Francisco, San Francisco, California, United States of America; 4 Department of Applied Biotechnology, Budapest University of Technology and Economics, Budapest, Hungary; Chinese University of Hong Kong, Hong Kong

## Abstract

**Background:**

Calpain proteases drive intracellular signal transduction via specific proteolysis of multiple substrates upon Ca^2+^-induced activation. Recently, dUTPase, an enzyme essential to maintain genomic integrity, was identified as a physiological calpain substrate in Drosophila cells. Here we investigate the potential structural/functional significance of calpain-activated proteolysis of human dUTPase.

**Methodology/Principal Findings:**

Limited proteolysis of human dUTPase by mammalian m-calpain was investigated in the presence and absence of cognate ligands of either calpain or dUTPase. Significant proteolysis was observed only in the presence of Ca(II) ions, inducing calpain action. The presence or absence of the dUTP-analogue α,β-imido-dUTP did not show any effect on Ca^2+^-calpain-induced cleavage of human dUTPase. The catalytic rate constant of dUTPase was unaffected by calpain cleavage. Gel electrophoretic analysis showed that Ca^2+^-calpain-induced cleavage of human dUTPase resulted in several distinctly observable dUTPase fragments. Mass spectrometric identification of the calpain-cleaved fragments identified three calpain cleavage sites (between residues ^4^SE^5^; ^7^TP^8^; and ^31^LS^32^). The cleavage between the ^31^LS^32^ peptide bond specifically removes the flexible N-terminal nuclear localization signal, indispensable for cognate localization.

**Conclusions/Significance:**

Results argue for a mechanism where Ca^2+^-calpain may regulate nuclear availability and degradation of dUTPase.

## Introduction

Calpains, the intracellular calcium activated cysteine proteases, play an important role in calcium-dependent signal transduction. Downstream protein targets are regulated by calpain-catalyzed limited cleavage, which usually results in a protein with modified activity. The potential role of calpain function in apoptotic pathways has been recently addressed in several laboratories [1], [2], [3], [4]. Calpain-induced degradation of numerous apoptotic or autophagy factors were implicated in switches between cell death pathways [5], [6], [7]. Identification of *in vivo* cellular substrates of calpain was attempted in a proteomic approach in *Drosophila* Schneider 2 cells, wherein we provided a list of putative *bona fide* targets [8]. One of these target proteins is dUTPase, lack of which is involved in inducing cell death [9], [10].

The enzyme dUTPase catalyzes the hydrolysis of dUTP to dUMP and pyrophosphate [11], [12]. The enzyme is a key regulator of cellular dUTP/dTTP pool ratios: on one hand, it removes dUTP from the DNA polymerization pathway, on the other hand, it contributes dUMP, the precursor for dTTP *de novo* biosynthesis [9], [13]. The function of dUTPase is essential to maintain genomic integrity; lack of the enzyme leads to massive uracil incorporation into the genome [14], [15], followed by repetitive futile cycles of repair resulting in DNA fragmentation and thymine-less cell death [16], [17]. Most dUTPases are homotrimers where the trimeric organization is indispensable for formation of active sites and catalysis [18], [19], [20], [21], [22]. In humans, two dUTPase isoforms exist, a nuclear (DUT-N, which is by far the major isoform in cycling cells) and a mitochondrial (DUT-M) form. The two isoforms are encoded on the same gene and are expressed under the control of alternate promoters [23]. Both forms contain localization signals on their N-termini; DUT-N possesses a nuclear localization signal (NLS) [24], while DUT-M has a mitochondrial leader peptide signal. The N-terminal segment of DUT-N is characfterized by high degree of conformational freedom. This segment has been shown to be prone to proteolysis in vitro by trypsin [21] and could not be located in the electron density map that led to determination of the three-dimensional structure of the enzyme in complex with the slowly hydrolysable substrate analogue α,β-imido-dUTP [21], [25].

In this study, we investigate the putative contribution of the calpain system to regulation of human dUTPase. We show that mammalian calpain catalyzes limited proteolysis of the nuclear isoform of human dUTPase. Identification of the specific cleavage sites by mass spectrometry reveals that this proteolysis results in removal of the nuclear localization signal at the N-terminus. Results indicate that Ca^2+^/calpain activation may perturb dUTPase localization and integrity, potentially leading to loss of dUTPase function within the nucleus.

## Results

### Calcium-dependent proteolysis of human dUTPase by calpain

**Figure 1:**
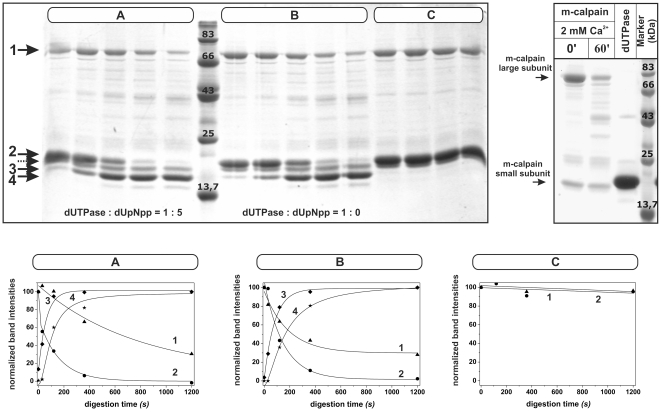
Calcium-dependent proteolysis of the human dUTPase. In vitro calpain cleavage was followed on a 15% SDS-PAGE. Panel (A): Samples correspond to the experiment in the presence of calcium and the substrate analogue. Panel (B): dUTPase was incubated with calpain in the presence of calcium, but without any substrate analogue. Panel (C): Control experiment was carried out in the absence of calcium. Incubation times were 0, 30, 120, 360, 1200 sec, respectively, in each sample except for the control, were time points were 0, 120, 360 and 1200 sec. Plots under the gel photographs represent changes in band intensities (line 1 (triangle) – calpain large subunit; line 2 (circle) – intact dUTPase; lines 3 (rhomboid), 4 (asterisk) – fragment forms of the dUTPase) against the time. Dotted arrow points at the small subunit of calpain. The intensity of the smeared band of the calpain large subunit in lane 1 (0 sec, panel A) is somewhat smaller than the intensity of the corresponding band in lane 2 (30 sec, panel A) - even if the smeared area is added to the densitometric evaluation - possibly due some leakage out of the leftmost well of the gel. To facilitate clear-cut identification of the bands corresponding to the calpain small subunit and intact human dUTPase that run very close to each other on the gel, the upper right panel shows calpain and dUTPase samples on their own.

To decide if human dUTPase may also be susceptible to calpain-catalyzed proteolysis, as we reported it in *Drosophila* [8], we prepared an in vitro digestion assay with the human recombinant DUT-N and rat m-calpain. The use of rat m-calpain instead of the human homologue may not cause any significant difference in this case as these two proteins share 94% identity and 99% similarity. We found that m-calpain can cleave dUTPase in the presence of calcium, but not in its absence (Fig. 1). During proteolysis, two distinct dUTPase fragments could be visualized (shown by numbered arrows 3 and 4). The presence of the substrate analogue α,β-imido-dUTP did not result in any significant alteration of the proteolytic process: no protective effect of α,β-imido-dUTP could be observed as followed on SDS-PAGE. This result suggests that the calpain cleavage sites may not be located in the vicinity of the substrate binding pocket.

**Figure 2:**
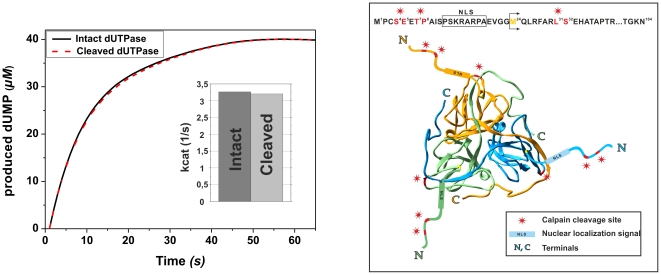
Mapping calpain cleavage sites. On the left panel, dUTPase activity was followed for the intact (solid line) and cleaved (dashed line) proteins. Bar graph represents catalytic rate constants. Right panel shows the 3D structure of the homotrimer dUTPase [16] (PDB: 3EHW) (ribbon model with the blue-, green-, and gold-colored monomers). The structure as observed in the electron density maps is schematically completed with a cartoon of the flexible N-terminals (M^24^, the first residue located in the electron density map is in yellow). Stars correspond to the calpain cleavage sites as determined by mass spectrometry.

To examine the possible changes in catalytic activity of the fragments compared to intact enzyme, we determined the catalytic rate constants of both intact and calpain-cleaved dUTPase forms. No difference in catalytic function was observed (Fig. 2), indicating that calpain digestion does not alter dUTPase activity.

### Identification of the calpain cleavage sites at the dUTPase N-terminal region

To identify the cleavage sites, fragments were analyzed by mass spectrometry. Three calpain-induced cleavage sites were identified in the two fragments separable on SDS-PAGE (bands 3 and 4 on Fig. 1). Table 1 provides a list of the peptides that were present in the samples, with highlighting those four peptides that were necessarily derived from calpain digestion as these contain non-tryptic sites. To ascertain the identity of these peptides, important for our study, we performed MS/MS analysis. The resulting spectra are shown in Figure S1, Figure S2, Figure S3 and Figure S4.

**Table 1:** List of peptides identified by mass spectrometry in lysates from gel bands 3 and 4 (cf Fig. 1).

m/z	z	Error/Da	Peptide	Start	End
***529.8700***	***2***	***0.098***	***(S)EETPAISPSK(R)***	***5***	***14***
***607.9600***	***2***	***0.14***	***(S)EETPAISPSKR(A)***	***5***	***15***
***428.3200***	***2***	***0.064***	***(T)PAISPSKR(A)***	***8***	***15***
434.3700	3	0.14	(R)ARPAEVGGM(Oxidation)QLR(F)	16	27
642.9900	2	0.14	(R)ARPAEVGGMQLR(F)	16	27
537.3800	2	0.11	(R)PAEVGGM(Oxidation)QLR(F)	18	27
529.3600	2	0.083	(R)PAEVGGMQLR(F)	18	27
361.6500	3	0.13	(R)LSEHATAPTR(G)	31	40
***485.3300***	***2***	***0.089***	***(L)SEHATAPTR(G)***	***32***	***40***
1092.6900	2	0.19	(R)AAGYDLYSAYDYTIPPM(Oxidation)EK(A)	45	63
723.5100	3	0.18	(R)AAGYDLYSAYDYTIPPMEK(A)	45	63
776.1300	2	0.24	(K)TDIQIALPSGC(Carbamidomethyl)YGR(V)	68	81
853.6000	2	0.19	(K)HFIDVGAGVIDEDYR(G)	92	106
626.0900	2	0.24	(R)GNVGVVLFNFGK(E)	107	118
502.0000	2	0.23	(R)IAQLIC(Carbamidomethyl)ER(I)	129	136
1034.2900	2	0.29	(R)IFYPEIEEVQALDDTER(G)	137	153

Peptides were generated by in-gel digestion of gel bands. Peptides with non-tryptic cleavage sites, generated by calpain digestion, are shown in bold italic font. The MS/MS spectra of these peptides are included in Fig. 2. Start and end positions are numbered according to the human dUTPase sequence. Residues in parentheses are the N- and C-terminal neighboring residues of the protein (not present in the peptides). Error indicates the difference between the measured and calculated peptide masses. Modifications are also noted. z, charge, m/z, mass normalized to charge.

Two calpain cleavage sites are very close together (^4^SE^5^; ^7^TP^8^), these two cleavage sites are found at the very beginning of the dUTPase sequence. There is no information in the literature about the function of this segment, which is not conserved among dUTPases.

The third calpain cleavage site is between the ^31^LS^32^ dipeptide that is located in the three-dimensional structure of human dUTPase (Fig. 2). Cleavage at this site results in a truncated protein that lacks the NLS segment (located between residues ^12^P-^19^A, [24]). The NLS segment shows conserved character with respect to e.g. Drosophila dUTPase where its lack was also shown to lead to exclusion of dUTPase from the cell nucleus [26]. Without this signal dUTPase is unable to enter the nucleus and may not function correctly.

### Cleavage of human dUTPase in HeLa cells via Ca^2+^-activation

**Figure 3:**
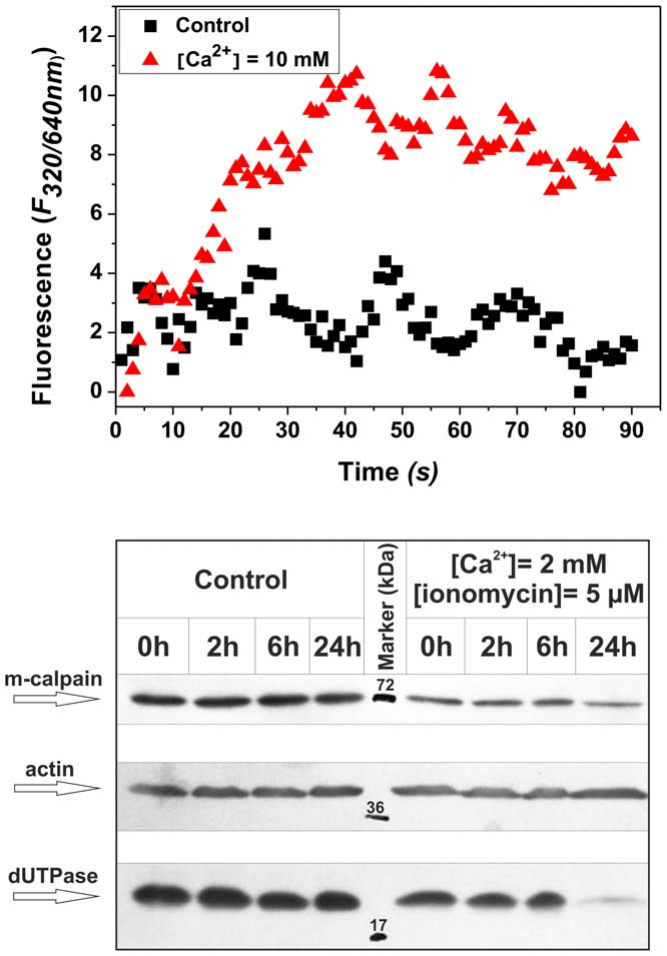
Degradation of cellular dUTPase pool upon calcium induction. Upper panel: The fluorescence intensity change corresponding to calpain activity in HeLa cellular extract was recorded in the absence of calcium (control, black squares), or the presence of 2 mM Ca^2+^ (red triangles). Bottom panel: HeLa cells were treated with ionomycin and calcium to activate their calpain pool. dUTPase degradation could be observed after 24 hours, but fragments could not be visualized, indicating potential further proteolytic events.

To test if the *in vitro* results may also be observed within intact cells, we followed dUTPase levels in HeLa cells in the absence or presence of Ca^2+^. In the latter case after 24 hours we observed significant dUTPase degradation, paralleled with some decrease in the m-calpain level also, while the dUTPase level in the control cells was unaffected (Fig. 3). However, we could not detect any dUTPase fragments, presumably because of further degradation processes. The slight change in the amount of m-calpain may be due to auto-proteolysis. As a control to monitor for equivalent amounts of total protein applied per lane, the Western blots were also developed against actin.

To directly demonstrate if the HeLa cell line contains a significant pool of Ca^2+^-activated calpains under our experimental conditions, we performed calpain activity measurements in HeLa cell extract in the absence and presence of Ca^2+^. Results show that the presence of Ca^2+^ led to calpain activation, indicating the presence of an active calpain pool (Fig. 3).

## Discussion

Intracellular proteolysis constitutes a main device of signaling. Identification of cognate proteolysis substrates in the specific pathways is indispensable to decipher the cellular network and significance of these pathways. Such investigations are particularly complex and demanding in the case of calpains that play roles in several pathways. As compared to digestive proteases like trypsin or caspases, calpains perform a more fine-tuned role by regulating the function of numerous substrates by limited proteolysis [27].

We have recently identified *in vivo* calpain substrates in a *Drosophila* system, providing a list of proteins, not yet described as calpain substrates, which belonged to several different cellular pathways, reflecting the well-known promiscuous calpain character [8]. In the present study, we focused on one of the hits, dUTPase, and wished to investigate if it is also a calpain substrate in a human model. Results of *in vitro* digestion experiments showed that human dUTPase is cleaved by Ca^2+^-calpain within the flexible N-terminus (Figs. 1, 2). Although catalytic function is unperturbed in the N-terminally truncated form (Fig. 2), cellular localization is expected to be drastically altered, since the truncated dUTPase species does not contain the NLS segment, indispensable for nuclear import [24]. Nucleo-cytoplasmic trafficking of trimeric dUTPases in eukaryotic cells depends on cognate peptide signals due to the approx. 50 kDa molecular mass of these proteins that prevents passive transport. Nuclear availability of the enzyme seems to be generally required for eukaryotic cells, attested to by the usual presence of a nuclear localization signal at the N-terminus [24], [26], [28]. Interestingly, for dUTPase from Singapore grouper iridovirus (a fish virus), a nuclear export signal was also described, indicating that trafficking of the host and the pathogen enzyme may be different [29].

The degradation of dUTPase was also observed in Ca^2+^-induced HeLa cells that showed an activated calpain pool (Fig. 3); however, we could not observe the fragments. As a speculative hypothesis, we propose that within the cells, calpain-cleaved dUTPase may be further degraded by other proteolytic events. Degradation of dUTPase in HeLa cells was only observed after 24 hours of Ca^2+^-induction, which, as we hypothesize, might be due to the fact that the cytoplasmic m-calpain may get hold of their nuclear substrates mainly during cell division when the nuclear envelope breaks down, and nuclear proteins gain even distribution throughout the cell. It is widely observed that calpains harbor several nuclear substrates [30], [31], and even play role in the regulation of cell division [32], [33], [34], [35].

**Figure 4:**
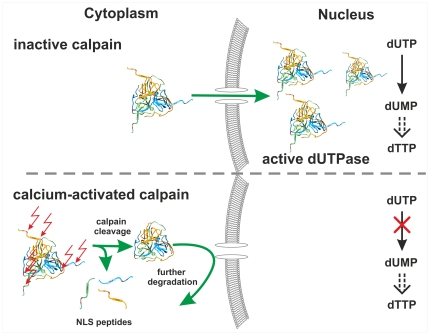
Schematic hypothetic model of the calpain effect on dUTPase. In cells where calpain is inactive, dUTPase (represented as the structural model in Fig. 2) can be imported into the nucleus and may catalyze the dUTP→dUMP conversion within the nuclear environment. Upon calcium-induction, calpain cleaves the N-terminal part of dUTPase (red arrows), resulting in an NLS-free enzyme. This truncated dUTPase, which may undergo further degradation, is unable to enter into the nucleus.

Based on the present results, we propose the hypothesis that calpain activation may control dUTPase localization and function through limiting its overall availability (Fig. 4). Upon Ca^2+^-induction, and following mitosis-coupled release of the nuclear dUTPase pool into the cytoplasm during the cell cycle, calpain may cleave the N-terminal of dUTPase removing the NLS segment. Truncated dUTPase is unable to enter the nucleus and may be further degraded. Lack of dUTPase function within the nucleus may lead to perturbed dUTP/dTTP ratios and provoke thymine-less cell death. Previously, several studies indicated that the level of dUTPase protein decreases during apoptosis [36], [37], [38]. Moreover, the potentially significant role of dUTPase as an anti-apoptotic protein has been recently suggested [39]. This pathway of inducing apoptosis by calpain action may act in parallel to other described roles of calpain in apoptosis (e.g. [1], [4]).

## Materials and Methods

### Preparation of recombinant proteins

Preparation of rat m-calpain was according to [40]. The human nuclear form of dUTPase (DUT-N) was expressed as a His-tagged protein and purified as described in [41]. Protein preparations were checked on SDS-PAGE and showed >95% purity. Protein concentrations were determined according to [42].

### In vitro digestion assays

Digestion assays were performed in 50 mM TRIS·HCl pH 7.50 buffer also containing 150 mM NaCl; 1 mM EDTA; 1 mM dithio-threitol; 30 µM human dUTPase and 2 µM rat m-calpain. Reaction was started by the addition of m-calpain in the presence or absence of 2 mM CaCl_2_. For digestion in the presence of substrate analogue α,β-imido-dUTP, the same reaction buffer was completed with 150 µM α,β-imido-dUTP and 10 mM MgCl_2_. Reaction was stopped with 3 mM EGTA after 40 minutes, digestion products were either directly submitted to mass spectrometry or separated on SDS-PAGE gels and then analyzed by mass spectrometry.

### dUTPase activity measurement

Enzymatic activity of both intact dUTPase and calpain-cleaved dUTPase was determined in steady-state pH indicator-based assays as described previously in [43], [44]. Reaction buffer contained 1 mM HEPES pH 7.50; 150 mM KCl; 40 µM Phenol Red indicator; 1 mM MgCl_2_; 40 µM dUTP and 150 nM dUTPase (from the digestion mixtures produced either in the absence or presence of Ca^2+^). The reaction was followed in 1 ml reaction volume thermostatted cuvette (25°C).

### Cleavage site identification by mass spectrometry

The calpain proteolysis cleavage products were desalted on C4 ZipTip (Millipore) and analyzed directly by MS on a Bruker Reflex III MALDI-TOF mass spectrometer in linear mode. Sinapinic acid was used as the matrix. External calibration was performed on the Bruker protein calibration standard I (#206355). Masses were determined by averaging seven independent measurements. For identification of calpain cleavage sites by MS/MS analysis, cleavage products were separated by SDS-PAGE, then gel bands were subjected to tryptic digestion followed by MS and MS/MS analysis as described in [8]. Database search was performed against the NCBI 20070629 protein database (5207057 sequences) using on-line Mascot search engine (version 2.2). Additionally, data were searched against our own database containing the His-tagged dUTPase sequence using in-house ProteinProspector server (version 5.3.0). For ion trap MS/MS data, monoisotopic precursor masses with peptide mass tolerance of ±1.2 Da and fragment mass tolerance of ±0.7 Da were considered. Semitryptic cleavages were allowed. Modifications considered: fixed - Cys carbamidomethylation; variable - acetylation of protein N-termini, methionine oxidation and pyroglutamic acid formation from N-terminal Gln residues.

### Cell culture and Western blotting

HeLa cells were obtained from Invitrogen. Cells were cultured in Dulbecco's Modified Eagle's Medium (DME) and Ham's F-12 Nutrient Mixture (Sigma) supplemented with Penicillin–Streptomycin solution (50 µg/ml; Gibco). The medium was complemented with CaCl_2_ in a final concentration of 2 mM. To induce calpain activation, 5 µM ionomycin (dissolved in DMSO) was added to the medium. Control cells were mock treated with DMSO alone. Cells were collected at indicated time points, washed twice with PBS, and resuspended in lysis buffer (50 mM TRIS·HCl pH 7.4; 140 mM NaCl; 0,4% NP-40; 2 mM DTT; 1 mM EDTA, 1 mM PMSF; 5 mM benzamidin, 1× complete™EDTA free protease inhibitors (Roche). Cell lysis was assisted by sonication five times (16 µ; 10 sec). Insoluble fraction was removed by centrifugation (20.000 g×15 min at 4°C). Protein concentration was measured with Bio-Rad Protein Assay to ensure equivalent total protein load per lane. Products were resolved under denaturing and reducing conditions on a 10 or 15% polyacrylamide gel and transferred to PDVF membrane. Western blot analysis was performed as below.

### Generation of dUTPase anti-serum and Western blotting

Experiencing non-specific character of commercially available antibodies against human dUTPase, we generated a new anti-serum following the procedure described in [45]. Briefly, rabbits were immunized with recombinant human dUTPase. Three immunising shots were given, at time intervals of 2–3 weeks, first in complete then in incomplete Freund's adjuvant or in physiological saline. Immunoblot analysis was done according to the method described previously [46]. Firstly, the membranes were reacted with rabbit serum containing polyclonal anti-dUTPase antibodies (used at 1∶5.000 dilution), or mouse anti-actin monoclonal antibody (Sigma-Aldrich), respectively. Secondly, the membranes were incubated with horseradish peroxidase conjugated secondary antibody: anti-rabbit IgG (Amersham Pharmacia Biotech) or antimouse IgG (Sigma), respectively.

### Calpain activity measurement in HeLa cell extract

To test the active calpain forms in HeLa extract, calpain activity was measured. Cells were collected and washed twice with 50 mM TRIS·HCl pH 7.50; 150 mM NaCl. To lyse the cells, they were resuspended in 50 mM TRIS·HCl pH 7.50 buffer containing 150 mM NaCl, 1 mM EDTA, 1 mM PMSF, and 5 mM benzamidin) and sonicated five times (16 µ; 10 sec). Insoluble fraction was removed by centrifugation (20.000 g×15 min at 4°C). Before the activity measurements, 2 mM 2-mercaptoethanol was added and the extract was incubated for 20 min on ice. Enzyme activity was measured with a modified FRET substrate as in [47], either in the absence or presence of 2 mM CaCl_2_.

### Chemicals

All chemicals were obtained from Sigma unless otherwise stated.

## Supporting Information

Figure S1 MS/MS spectrum of m/z 529.87 (2+) confirming peptide sequence EETPAISPSK. # stands for water loss.
(TIF)

Figure S2 MS/MS spectrum of m/z 607.96 (2+) confirming peptide sequence EETPAISPSKR. # stands for water loss.
(TIF)

Figure S3 MS/MS spectrum of m/z 428.32 (2+) representing peptide sequence PAISPSKR. # stands for water loss.
(TIF)

Figure S4 MS/MS spectrum of m/z 485.33 (2+) confirming peptide sequence SEHATAPTR. # stands for water loss.
(TIF)
