# Supplementary figures and images for: Calpain-Catalyzed Proteolysis of Human dUTPase Specifically Removes the Nuclear Localization Signal Peptide
Source: PLoS One. 2011 May 19;6(5):e19546. doi: 10.1371/journal.pone.0019546 (PMC3098232; doi:10.1371/journal.pone.0019546)

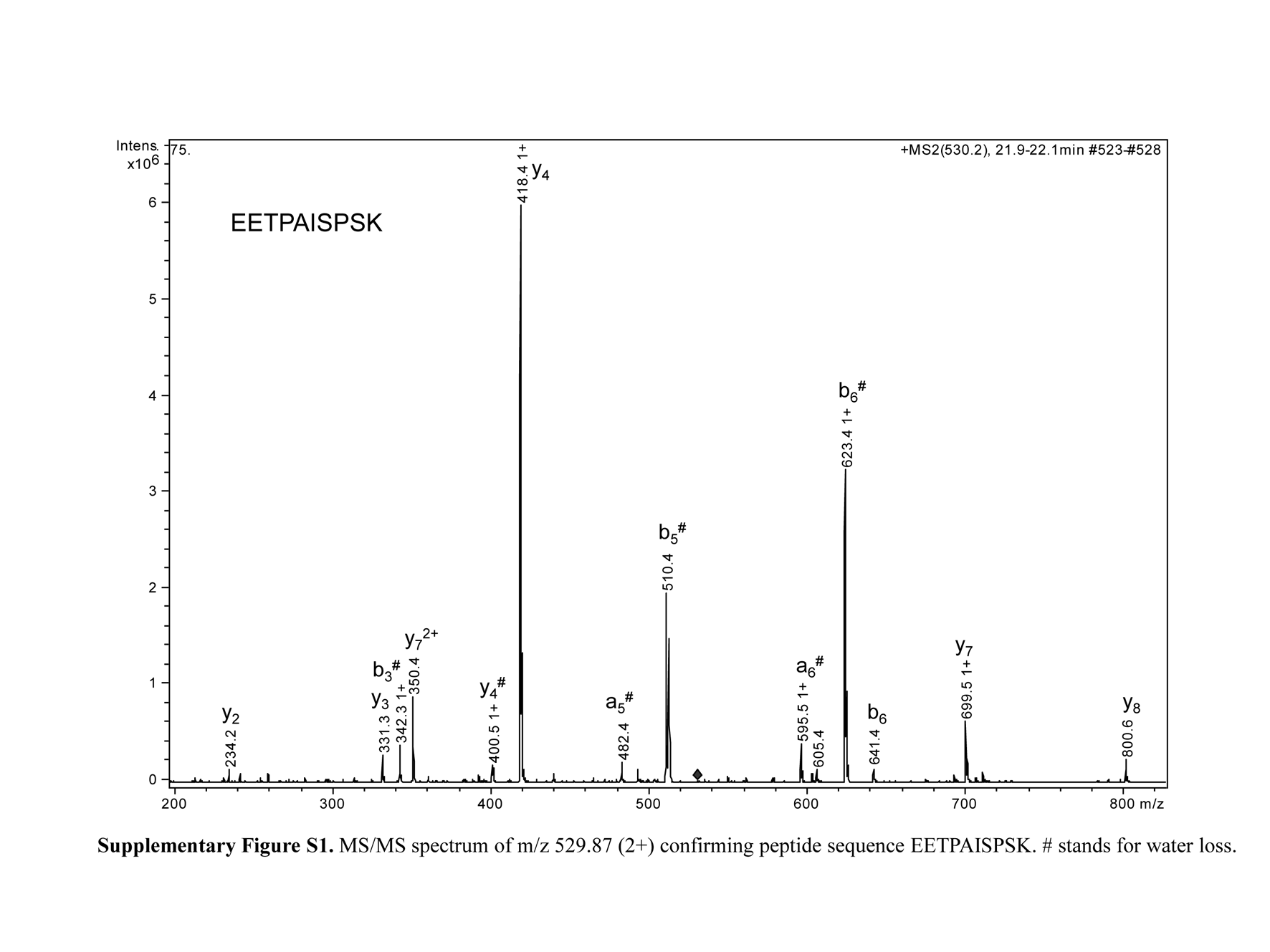

Supplement: Figure S1 — MS/MS spectrum of m/z 529.87 (2+) confirming peptide sequence EETPAISPSK. # stands for water loss. (TIF) [file pone.0019546.s001.tif]

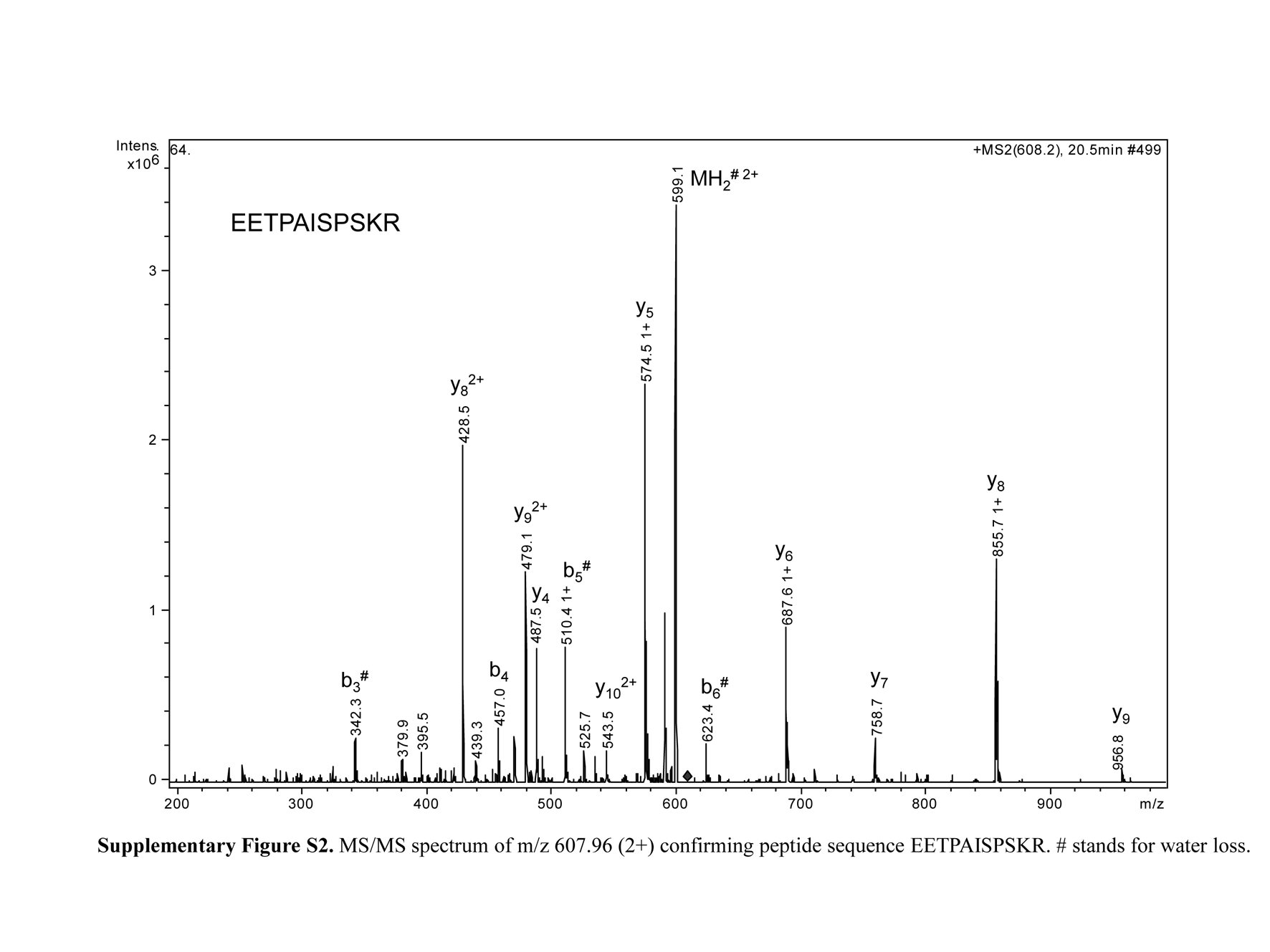

Supplement: Figure S2 — MS/MS spectrum of m/z 607.96 (2+) confirming peptide sequence EETPAISPSKR. # stands for water loss. (TIF) [file pone.0019546.s002.tif]

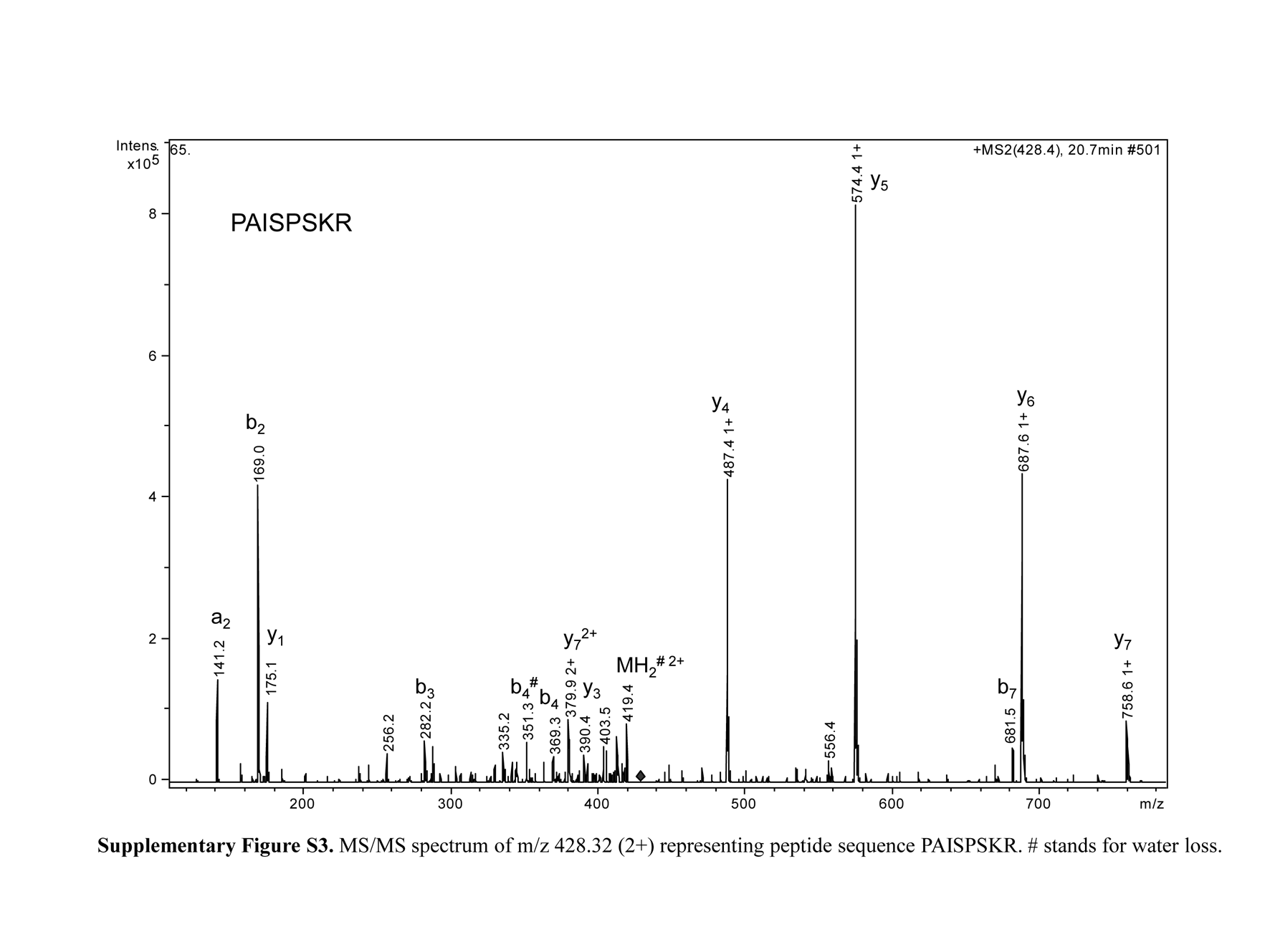

Supplement: Figure S3 — MS/MS spectrum of m/z 428.32 (2+) representing peptide sequence PAISPSKR. # stands for water loss. (TIF) [file pone.0019546.s003.tif]

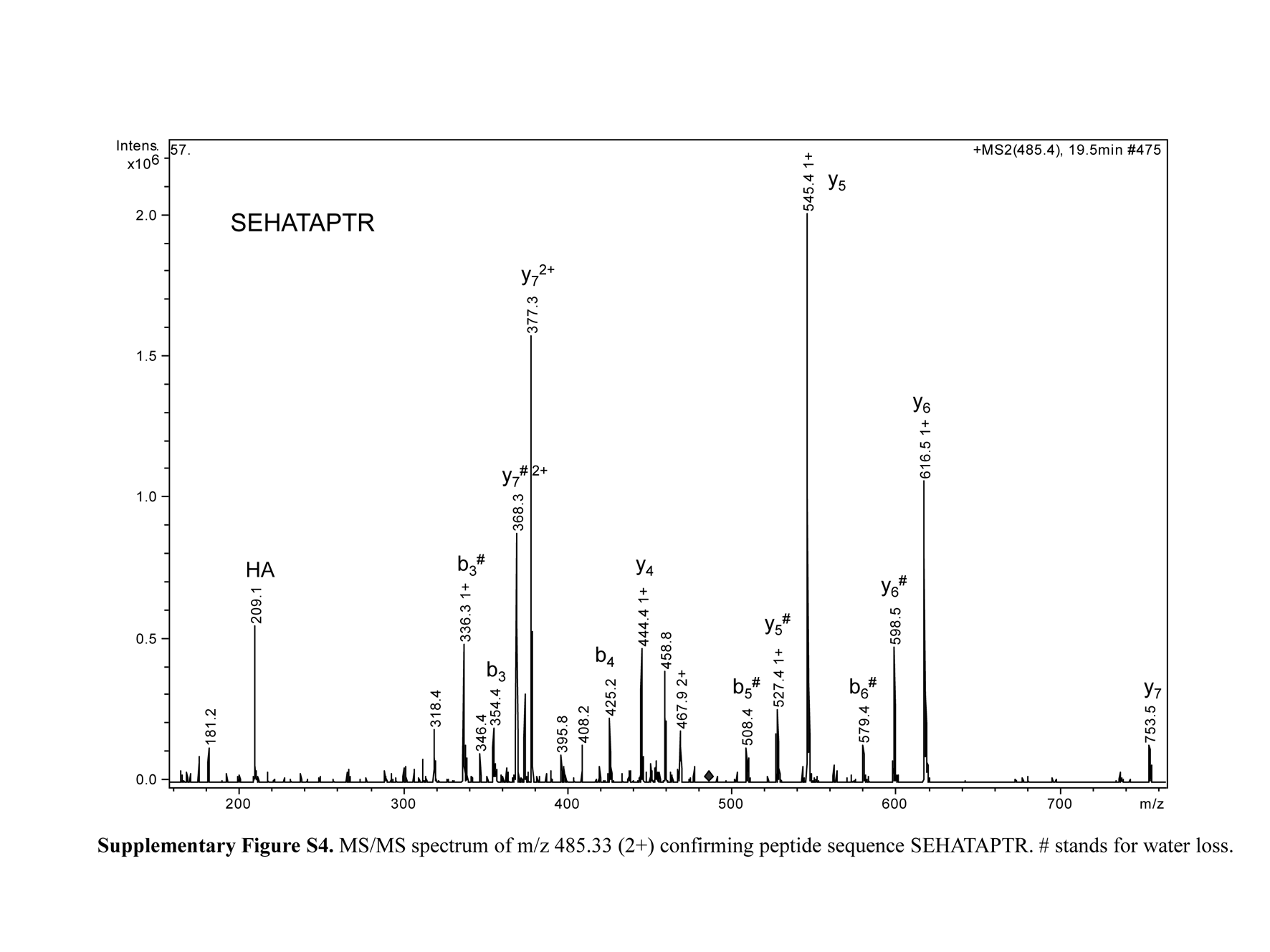

Supplement: Figure S4 — MS/MS spectrum of m/z 485.33 (2+) confirming peptide sequence SEHATAPTR. # stands for water loss. (TIF) [file pone.0019546.s004.tif]
